# Exploring Suicide-Related Internet Use Among Suicidal Mental Health Patients in the United Kingdom: Cross-Sectional Questionnaire Study

**DOI:** 10.2196/70458

**Published:** 2025-07-08

**Authors:** Lana Bojanić, Isabelle M Hunt, Sandra Flynn, Saied Ibrahim, Pauline Turnbull

**Affiliations:** 1 Division of Psychology and Mental Health, University of Manchester, Oxford Rd, Manchester, M13 9PL, United Kingdom, 44 01612750709

**Keywords:** mental helath patients, suicide, internet, online searches, online survey

## Abstract

**Background:**

The dual nature of suicide-related internet use (SRIU) as preventative or harmful is well-documented, but its characteristics in the mental health patient population remain underresearched. Some evidence suggests mental health patients engage in SRIU differently from the general population.

**Objective:**

This study aims to explore the types, motivations, frequency, and perceived impacts of SRIU in suicidal mental health patients, as well as their engagement with web-based prevention resources.

**Methods:**

A cross-sectional study was conducted using an anonymous web-based survey distributed between June and December 2023. Participants (n=696) were UK adults with secondary mental health service contact and recent suicidal thoughts or behaviors. Of these, 523 (75%) participants engaged in SRIU. Collected data included sociodemographic details, clinical history, types and motivations for SRIU, and interactions with suicide prevention resources. Analysis used descriptive statistics, chi-square, and Wilcoxon tests, with multiple testing corrections applied.

**Results:**

The most common SRIU type was searching for suicide-related content (456/523, 87.4%), followed by connecting with others (271/523, 51.8%). Motivations included seeking information on suicide methods (313/523, 60.8%) and support for suicidality (271/523, 57.2%), with significant overlap. Participants perceived SRIU as neither harmful nor helpful overall, with those seeking suicide methods rating it as more harmful. Most participants encountered suicide prevention messaging, but less than half engaged with it. Only 27.5% (n=144) participants disclosed their SRIU to clinicians, with only 1 in 10 being asked about it by their clinician.

**Conclusions:**

This study underscores the dual role of SRIU as both a source of support and a potential risk for mental health patients. Despite high exposure to suicide prevention messaging, engagement was limited, suggesting inefficiencies in current intervention designs. Clinicians rarely inquired about SRIU, and voluntary disclosure by patients was low, representing missed opportunities for intervention. Proactive discussions about SRIU in clinical settings could improve risk identification and support planning. Addressing its harmful aspects while leveraging its potential for support requires integrated online and offline strategies.

## Introduction

Studies have shown that people who struggle with suicidality can feel empowered by accessing information via the web, enabling them to seek support, access help resources, and connect with others who may be experiencing similar issues [1]. Nevertheless, using the internet for suicide-related reasons is not without risks and can facilitate access to information on suicide methods and reinforce suicidal thoughts and self-harm [2]. This dual nature of suicide-related internet use (SRIU) is typically explained in the literature by whether the content is perceived by the suicidal person to be helpful (preventative) or harmful (prosuicide). However, categorizing behaviors related to accessing suicide-related content (SRC) into prosuicide and preventative categories has not been shown to predict users’ suicidal behavior or ideation [3,4]. Additionally, research suggests that people who use the internet when suicidal rarely access just one type of content, with a UK study showing most young people who accessed prosuicide sites also accessed suicide prevention sites [5]. SRIU is a complex behavior that cannot be easily categorized as purely harmful or preventative, nor can its impact on a person’s suicidal thoughts and behaviors be reliably predicted.

Evidence suggests that those in contact with mental health services may engage in SRIU in a different way than those in the general population. A UK study found that those admitted to hospital for self-harm had primarily focused on researching suicide methods and purposefully avoided helpful resources in contrast to those in the general population who often accessed suicide prevention content [6]. Mental health patients often turn to the internet to fill gaps in service provision by researching their illness and medications, while also frequently finding support within web-based communities to counteract the marginalization and stigmatization they may experience [7,8]. Given the specifics of SRIU in mental health patients, discussing their internet use with clinicians could inform prevention efforts by identifying and recognizing risk factors, as well as provide insights into their suicidality. However, recent qualitative research indicated that clinicians rarely inquire about SRIU, relying instead on patients to self-disclose this behavior [9]. This puts the burden of disclosure on the patient and, if they are already struggling, underscores the need for clinicians to take a more proactive approach.

Web-based prevention efforts are equally needed to curb potentially harmful aspects of SRIU. According to a Google blog, since 2010 Google’s “suicide prevention result” (SPR, also known as Google OneBox), most commonly a banner with a suicide hotline number presented after suicide-related search results, has been considered a first line of defense for harmful SRIU [10]. Additionally, the World Health Organization’s web-based media guidelines suggest the inclusion of information on where and how to seek help for suicidal thoughts within suicide-related media content [11]. However, there is a paucity of evidence on whether people, who use the internet for suicide-related purposes, including mental health patients, contact advertised helplines or use other prevention resources when engaging in this behavior [12].

More studies are needed that directly recruit people who use the internet when suicidal to understand the motivation, frequency, and outcomes of their SRIU [13] and this is especially true in mental health patient populations. The aim of this study was to describe the types of and motivations behind SRIU of suicidal mental health patients, its frequency, perceived helpfulness, and any engagement with web-based suicide prevention resources.

## Methods

### Study Design

This study used a cross-sectional design using an anonymous web-based survey hosted on Qualtrics (Qualtrics International Inc) between June 1, 2023, and December 31, 2023.

The 45-question survey (available in the Multimedia Appendix 1), developed with input from coauthors and members of a patient and public involvement group, was split into four sections: (1) sociodemographic details, (2) clinical history, (3) suicidal thoughts and behaviors, and (4) SRIU. Section 4 comprises questions on the type of and motivation for SRIU, frequency and perceived helpfulness/harmfulness of SRIU, and interaction with Google’s “suicide prevention result.” Participants could select more than one type of and more than one motivation for SRIU (Textbox 1). Furthermore, participants were asked whether they searched for specific suicide-related sites, knowledge of safe internet use when engaging in SRIU, and whether they had disclosed their use to their clinician; this was captured using multiple choice questions. There were 4 free-text questions relating to search terms used to look for suicide-related information, details of any disclosure of this behavior to their clinician, and any additional thoughts on SRIU participants might have had; qualitative findings from this free-text data will be presented elsewhere. The survey also included visual analog scales (VASs) for questions on the perceived helpfulness/harmfulness of SRIU in general and of SPRs. VAS scores ranged from 0 to 10 and were interpreted as follows: 0‐2: very helpful; 2.1‐4.0: moderately helpful; 4.1‐6.0: neither helpful nor harmful; 6.1‐8.0: moderately harmful; and 8.1‐10.0: very harmful.

Textbox 1. **Types and motivation for suicide-related internet use (SRIU). Participants were able to select multiple responses.**

Types of SRIU

- Searched for suicide related content/information (eg, browsing the internet or social media) because of your own suicidal thoughts and feelings.
- Created suicide related content/information (eg, blogging or posting pictures).
- Engaged with (eg, commented or reposted) suicide-related.
- Accidentally came across suicide-related content while browsing internet/social media.
- Used the Internet to interact/connect with others because of your own suicidal thoughts and feelings.
- None of the above.

Motivations for SRIU

- Seeking information on support/help for my own mental health (eg, Samaritans, NHS [National Health Service] web pages).
- Seeking information on methods of suicide/harming oneself.
- Seeking support from friends/peers.
- Interacting with celebrity bloggers/influencers.
- Engaging in discussions about suicide/harming oneself (eg, forums, Twitter).
- Sharing personal experiences of suicidality (eg, on social media).
- Researching medication (eg, side effects) that you have been taking for your mental health.
- Curiosity about suicide.
- Raising awareness or campaigning for suicide prevention.

### Procedure

A list of resources such as helplines and services participants could contact if they became distressed was provided. The first 2 sections covered sociodemographics and mental health history. The third section covered suicidal thoughts and attempts, adverse life events (ie, relationship breakdown and financial difficulties) that may have contributed to their suicidality, and perceived support received from friends, family, and their community. Trigger warnings for questions related to suicidal thoughts and behaviors and the opportunity to skip them were provided. The fourth section covered SRIU defined as: “Suicide-related internet use is any internet use related to your thoughts, feelings and behaviors connected to suicide. This kind of use can be, for example, expressing suicidal feelings or thoughts on social media or using the internet to get help and support when feeling suicidal.*”* Participants were asked if they engaged in SRIU in the past 12 months and if they answered “no,” they were exited from the questionnaire. Those who answered “yes,” continued with section 4 on SRIU. All participants were provided with a list of helplines and services on exiting the survey.

### Recruitment

Eligibility criteria included adults aged 18 years or older, a history of suicidal thoughts or behaviors within the past 12 months, living in the United Kingdom, and contact with secondary mental health services. In the United Kingdom, secondary mental health services provide specialized care for people with severe and complex mental health conditions. Participants were recruited via the web and on radio; via social media (X, Reddit, Facebook, BlueSky, Instagram, Mastodon, TikTok, and LinkedIn); and via mailing lists, web pages, and newsletters of charities and the University of Manchester, with the recruitment materials often organically shared throughout and between platforms.

### Participants

Of 1057 initial respondents, 72 (6.7%) respondents did not answer questions pertaining to inclusion criteria, and 188 (17.8%) respondents did not meet the inclusion criteria. Specifically, 110 out of 188 (58.5%) respondents had no contact with secondary mental health services, 43 (22.8%) respondents did not report suicidal thoughts or behavior, and 35 (18.6%) respondents failed to meet both inclusion criteria. A further 23 respondents met the inclusion criteria but did not answer any of the questions. The survey also included one attention-check question and data were manually checked for joking or aberrant answers to ensure data integrity; 3 participants were excluded for failing the attention-check question, and a further 5 for aberrant or joking answers. There were no fully duplicated responses (ie, no evidence that the very same answers had been submitted multiple times). Finally, 70 participants did not provide information on whether they had used the internet for suicide-related purposes. In total, 696 participants met the inclusion criteria and completed the survey, and 523 (75%) participants indicated they had engaged in SRIU. Participants who reported only encountering SRC accidentally (n=50) were grouped with those who did not engage in SRIU, as they did not actively seek out such content.

### Statistical Analysis

Descriptive analysis of categorical data was conducted using frequencies and proportions. The denominator for each estimate was the number of valid cases for the specific variable, meaning cases with missing information were excluded from the analysis of that variable. Percentages of missing values for each variable are presented in Table 1. For data collected via VASs, the median and IQR were used for analysis, as recommended by Heller et al [14]. Different types and overlaps of types of motivations for SRIU have been visualized using an UpSet diagram. An UpSet diagram displays the sizes of individual sets alongside their intersections. The plot includes a matrix of dots indicating intersecting sets, with a bar chart above showing the size of each intersection and a bar chart to the left showing the total size of each set. Chi-square tests were used for comparisons of categorical variables, while the Wilcoxon test was used for comparisons of VAS data. To account for multiple testing, the Bonferroni correction was applied to *P* values, with values below .001 (.01 divided by the number of comparisons) considered statistically significant. All analyses and figure generation were performed using RStudio (RStudio Team, 2022).

**Table 1.** Missingness pattern of analyzed variables.

	Missing values, n/N (%)
Type of SRIU^a^	0 (0)
Motivation for SRIU	25/523 (4.8)
Searching for specific suicide-related websites/forums	23/523 (4.4)
Frequency of SRIU	23/523 (4.4)
Perceived helpfulness/harmfulness of SRIU	28/523 (5.4)
Encountering SPR^b^ online	21/523 (4.0)
Interacting with SPR	8/425 (1.9)
Perceived helpfulness/harmfulness of SPR	3/193 (1.6)
Knowledge about safe internet use while engaging in SRIU	30/523 (5.7)
Clinician’s enquiry about SRIU	30/523 (5.7)
Participant’s disclosure to the clinician about SRIU	30/523 (5.7)

a SRIU: suicide-related internet use.

b SPR: suicide prevention result.

### Ethical Considerations

Ethics approval for the study was granted by the University of Manchester Research Ethics Committee (ref: 2023-16133-28055). Before commencing the questionnaire, participants were presented with the study information sheet and informed consent was obtained. Participants were not offered any financial incentives, such as monetary compensation or vouchers, for their participation in this study. Finally, participants were not asked to disclose any identifiable data (ie, name, email address, geographical location) nor were their IP address data collected.

## Results

### Characteristics of Participants Who Have Engaged in SRIU

In total, 523 participants had engaged in some type of SRIU in the past 12 months. While a forthcoming study using the same dataset will examine the demographic and clinical characteristics of these individuals in greater detail, we will briefly present their key characteristics here. Of those who have engaged in SRIU, 64% (n=337) were female, and most were aged between 25 and 34 years (239/523, 46%). The majority were White (459/523, 88%), and more than half identified as nonheterosexual (304/523, 58%). Gender identity differed from birth sex for 16% (n=85) of the participants, and most had higher education qualifications, with 56% (n=291) holding a university degree or higher.

Regarding clinical characteristics, the most common psychiatric diagnoses, both in the last year and historically, were affective disorder and anxiety disorders. Most participants (424/519, 81%) had been prescribed psychotropic medication in the last year and 18% (n=95) had been admitted for psychiatric in-patient care. Most participants were moderately dissatisfied with their mental health service provision.

**Figure 1.**
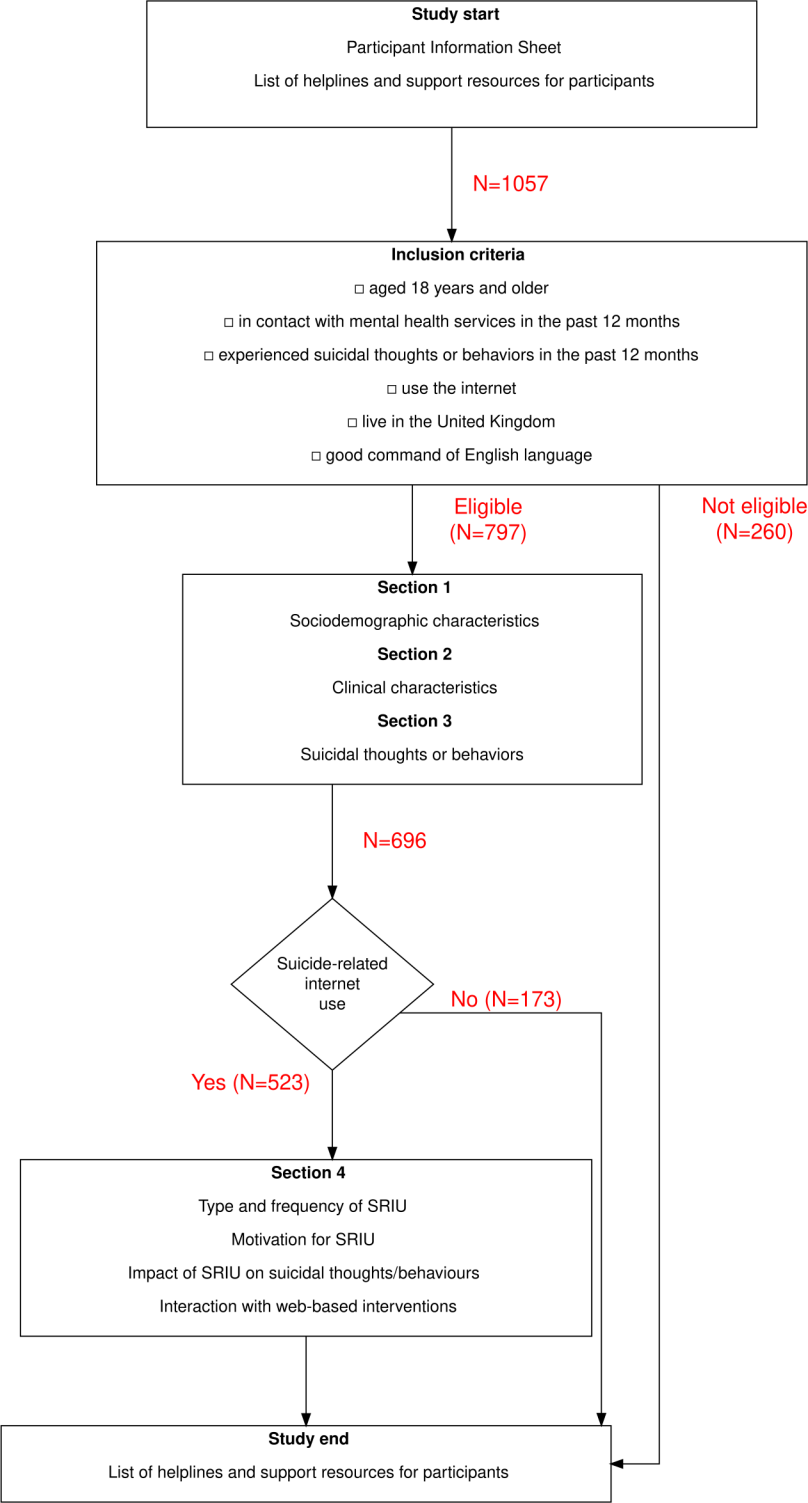
Study flowchart: inclusion criteria and sections of survey. SRIU: suicide-related internet use.

Participants who engaged in SRIU reported moderately high-intensity of suicidal thoughts, 35% (n=183) of them daily. Furthermore, 88% (n=459) participants reported disclosing these thoughts to someone, most likely to mental health professionals (364/523, 70%). One-third (170/523) of the participants had attempted suicide in the last year with moderately high planning involved and 34% (57/158) participants disclosed the plans of this attempt to someone. Life stressors, including relationship difficulties (38%, 179/471), financial problems (152/471, 32%), and insomnia (155/471, 33%) were also common among participants and they reported moderately low support from family and friends. Figure 1 depicts the survey sections and flow.

### Types and Motivations for SRIU

**Figure 2.**
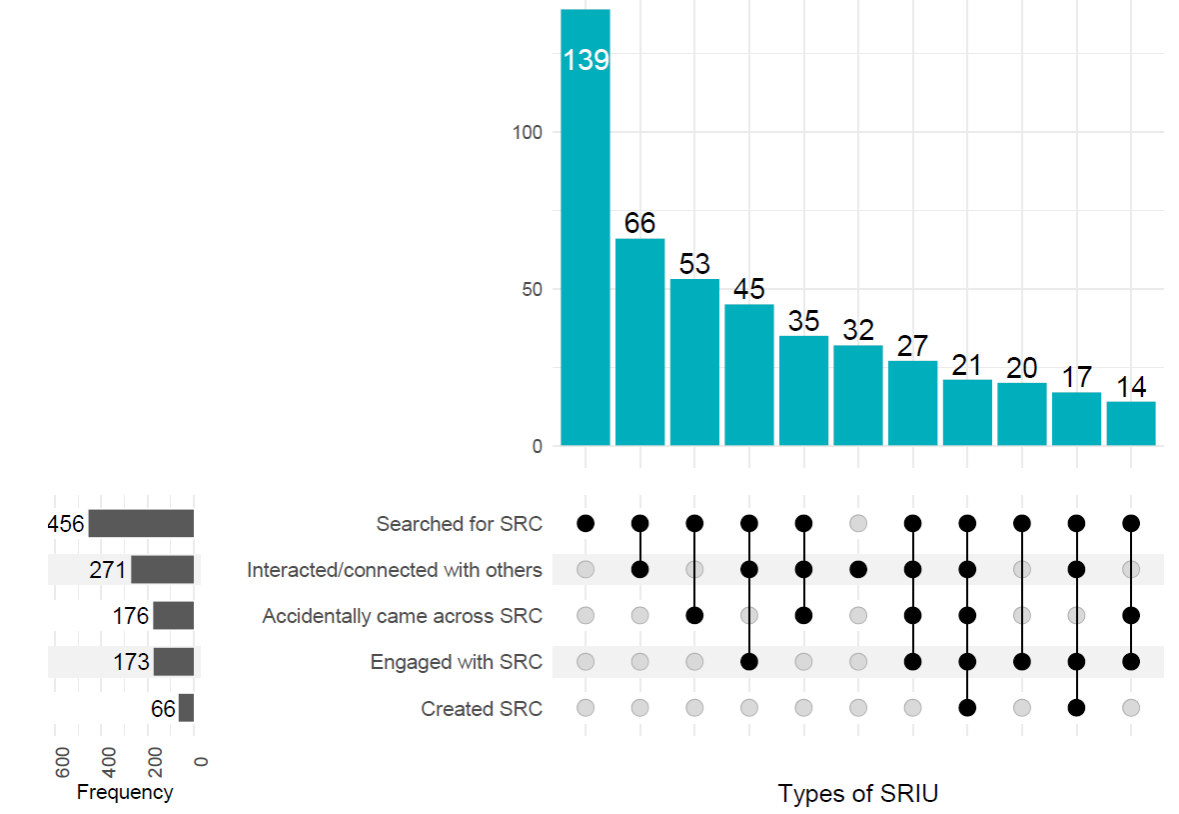
UpSet diagram representing frequency and overlap between different types of SRIU of mental health patients (n=523) (Overlaps <8 have not been shown; therefore, the number in the figure does not total 523). SRC: suicide-related content; SRIU: suicide-related internet use.

The different types of and motivations for engaging in SRIU are shown in Figures2  3. These were not mutually exclusive, meaning participants could select multiple types of SRIU as well as multiple motivations.

Two-thirds of participants (345/523, 67.7%) engaged in more than one type of SRIU, with 21 (4%) participants having engaged in all types (Figure 2). The most common type was searching for SRC (456/523, 87.4%), and the second most common was interacting or connecting with others for reasons relating to the participants’ suicidality (271/523, 51.8%). A third (173/523, 33.1%) of the participants had engaged with (ie, commented and reposted) and 66 (12.6%) participants had created SRC themselves. Another third (176/523, 33.7%) of participants also came across SRC accidentally while browsing the internet.

A third (32.3%) of participants reported only engaging in one type of SRIU in the past 12 months. Of these, the vast majority (139/178, 78.7%) primarily searched for SRC, representing 26.4% of all participants. The remaining participants who only engaged in one type of use (32/178, 18.0%) engaged solely in interactions or connections with others due to their suicidality.

**Figure 3.**
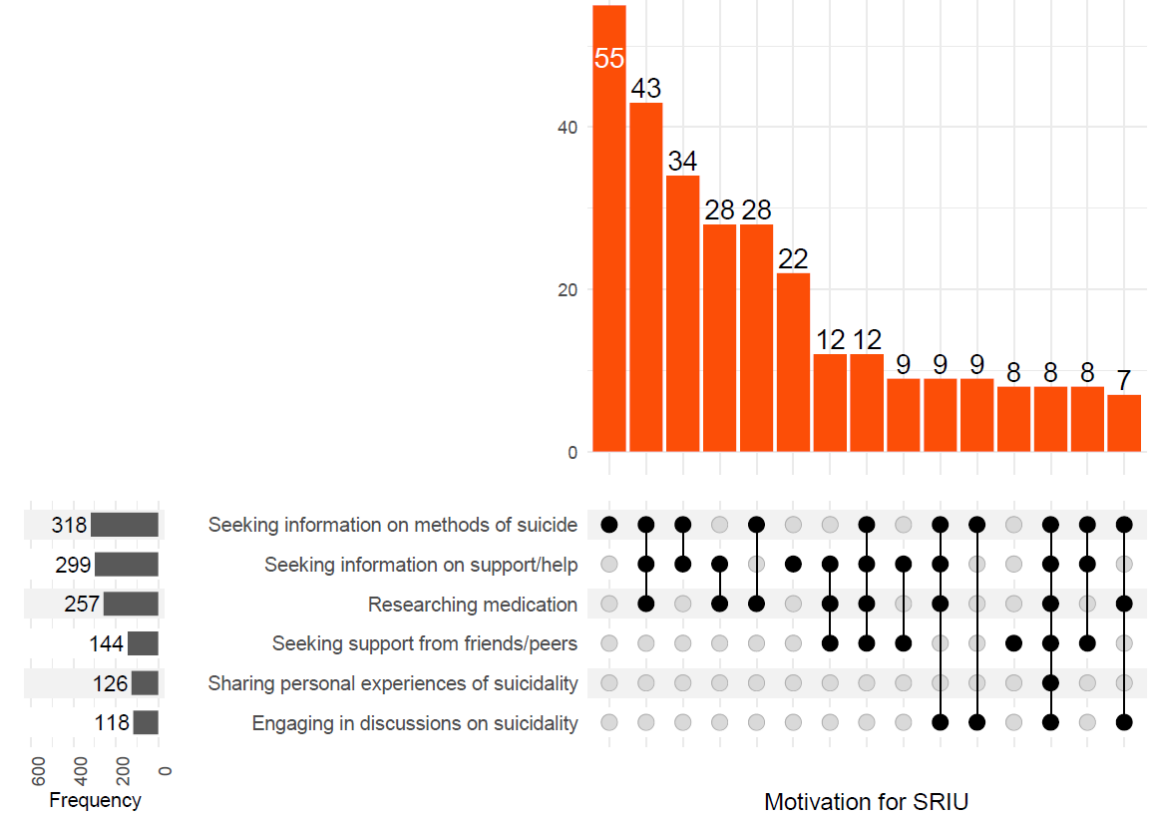
UpSet diagram representing frequency and overlap between different motivations for SRIU of mental health patients (n=523) (Overlaps <8 have not been shown; therefore, the number in the figure does not total 523). SRIU: suicide-related internet use.

Similarly, the majority of participants had more than one motivation to engage in SRIU (397/523, 75.9%). The most common motivations were seeking information on methods of suicide (318/523, 60.8%) and seeking information on support or help for one’s own suicidality (299/523, 57.2%; Figure 3). Other common motivations were researching medication that participants were taking (257/523, 49.1%), seeking support from friends and peers for one’s suicidality (144/523, 27.5%), sharing personal experiences of suicidality (126/523, 24.1%), and engaging in discussions about suicide (118/523, 22.6%). The most common combination of motivations to engage in SRIU was seeking information on both help and support as well as suicide methods and researching medication (43/523, 8%; Figure 3).

Of the 101 (19.9%) participants who only reported one motivation to engage in SRIU, most were only motivated to search for suicide methods (55/101, 54.5%), 11% of all participants. A further 22 (21.8%) were only motivated to search for helpful resources, 4% of all participants. Notably, 25 participants (4.8%) did not report any motivation to engage in SRIU.

**Figure 4.**
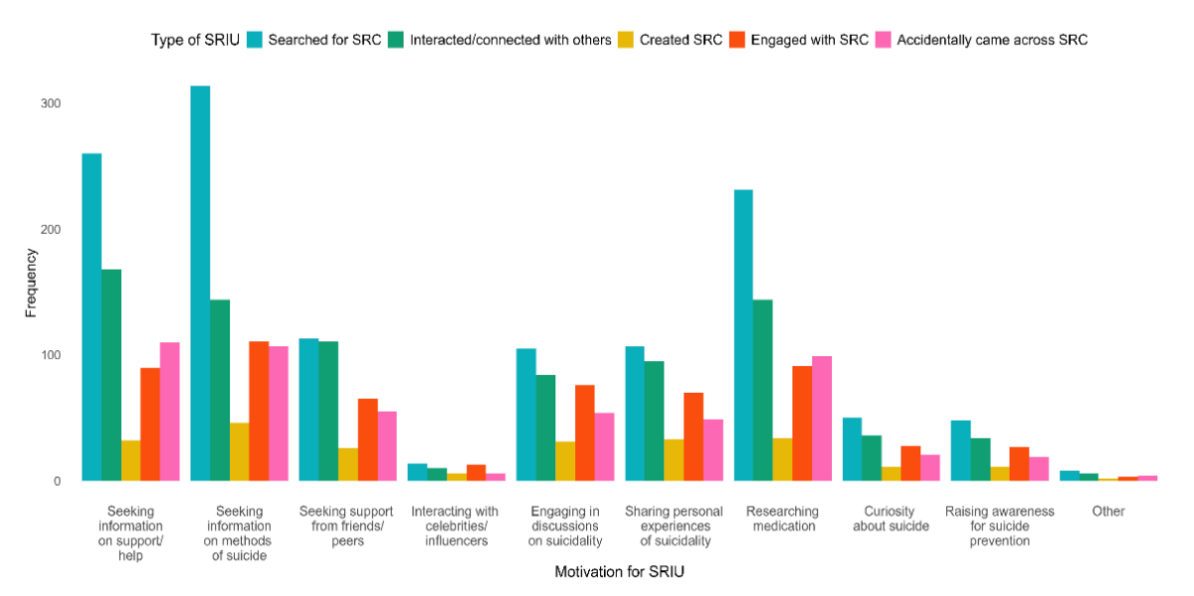
Types of and motivations for SRIU (n=523). SRC: suicide-related content; SRIU: suicide-related internet use.

Figure 3 shows the interplay of types of SRIU with motivation for engaging in it; a participant could choose multiple types of SRIU and multiple motivations for it. Regardless of the motivation, searching for SRC was the most common type of SRIU participants engaged in. Interacting with others for reasons relating to one’s own suicidality was a common type of SRIU in those who were motivated to seek support from friends or peers and to share personal experiences of suicidality (Figure 4).

### Characteristics of SRIU

In the whole sample, the average perception of SRIU was neither helpful nor harmful (VAS score median 5, IQR 4.1‐6.1). Those who searched for SRC perceived their SRIU as somewhat more harmful than those who did not (VAS score median 5.0, IQR 4.4‐6.4 vs VAS score median 4.9, IQR 3.3‐5.0; *P*<.001). There was no difference in perceived harm/helpfulness for any other type of SRIU. Furthermore, those who were motivated to engage in SRIU by seeking suicide methods perceived their internet use as somewhat more harmful compared with those who were not motivated by this (VAS score median 5.0, IQR 4.9‐6.6, vs VAS score median 5.0, IQR 3.5‐5.3; *P*<.001); again, there was no difference in perceived harm/helpfulness for any other motivation for SRIU. Among participants, 43.2% (n=216) used the internet for suicide-related purposes at least once a week.

Most participants did not search for specific suicide-related web pages, groups, or profiles (397/500, 75.9%). Participants who had engaged with SRC were more likely to search for specific sites compared with those who did not (51.5% vs 27.7%, *P*<.001). In terms of motivation, those who were motivated by seeking information on suicide methods and by wanting to discuss suicidality were more likely to search for specific sites compared with those who did not (79.6% vs 58.9%, *P*<.001; and 37.9% vs 19.4%, *P*<.001 respectively). Less than half of the participants (241/493, 46.1%) who had used the internet for suicide-related purposes knew where to find information on how to use the internet safely.

While engaging in SRIU, most participants had seen suicide prevention messaging (425/502, 84.7%) and nearly half (193/425, 45.4%) had interacted with these messages, including following links to helpful content or calling the helpline number. Participants who searched for SRC were more likely to see suicide prevention messaging (89.9% vs 72.7%, *P*<.001), and those who were motivated to seek help via the web were more likely to engage with these interventions compared with those who were not motivated to seek help via the web (73.6% vs 49.6%, *P*<.001). Those who interacted with suicide prevention messaging found them neither helpful nor harmful (VAS score median 5, IQR 4.0‐7.0) and there was no difference in the perceived harmfulness/helpfulness of these interventions regarding the type of or motivation for SRIU.

Most participants had not been asked by a clinician about their SRIU (466/523, 89.1%). Around a quarter (144/523, 27.5%) self-disclosed SRIU to their clinician. There was no difference in disclosure regarding the type of or motivation for SRIU.

## Discussion

### Main Findings

This study, conducted in the United Kingdom, found the most common form of SRIU in our sample was searching for SRC via the web, with 87.4% (n=456) of participants engaging in this behavior. A quarter engaged in searching for SRC only without other types of SRIU. Additionally, 51.8% (n=271) of participants interacted with others via the web about their suicidality, and 12.6% (n=66) created SRC. We found that common motivations for engaging in SRIU included seeking information on suicide methods (318/523, 60.8%) and finding support for their own suicidality (299/523, 57.2%). Participants who searched for SRC were more likely to view it as harmful, particularly if motivated by a desire to learn about suicide methods. Less than half of participants who have seen web-based suicide prevention messaging choose to engage in it and have perceived it as neither helpful nor harmful.

Previous epidemiological studies have shown moderate associations between web-based searches for SRC and deaths by suicide [15-17]. A high prevalence of searching for SRC was also found among mental health patients who either attempted or died by suicide [6,18-20]. In our study, it was possible to delineate the type of SRIU by motivation. The most common motivations for engaging in SRIU were searching for help and searching for suicide methods; this duality aligns with a recent study on a clinical suicidal population which noted that patients include the internet as both a potentially helpful factor and a harmful one in their self-made safety plans [21]. A systematic narrative review on web-based mental health help-seeking in young people also found that web-based searching was the most common help-seeking behavior, followed by participation in web-based communities and discussion forums [22], as was the case in our study. Research on mental health discussion forums has shown that most participants join suicide-related discussions to seek help from others who they feel understand them and that these interactions can reduce suicidality [8,23]. Additionally, our results show that mental health patients rarely have only one motivation for SRIU; however, when this was the case, they were more likely to be motivated to search for information on suicide methods than for help. Researching suicide methods via the web is almost always considered harmful by researchers and clinicians since it can expose people to detailed information on how to harm themselves which can increase the risk of suicide [24].

In previous research conducted by this team [9,18], obtaining information on suicide methods via the web was the most common type of SRIU in mental health patients who died by suicide, and also the most common type of SRIU patients disclosed to clinicians. We hypothesized that this is likely to have occurred because the disclosure of research methods via the web naturally happens during risk assessments when clinicians ask patients about their suicide planning. This combination of the high prevalence of actively searching for SRC via the web and motivation to seek information on suicide methods was self-reported by suicidal mental health patients in this study. Taken together, the evidence suggests the significant role that web-based searches, especially those concerning methods of suicide, can play in the suicidality of mental health patients, especially in the choice and potential lethality of suicide methods. Our findings also suggest that participants who searched for SRC, particularly those motivated to search for methods, perceived their SRIU as more harmful on average. This finding may be explained in 2 ways: first, searching for SRC, especially information on suicide methods, might indicate higher suicidality and thus higher distress; second, exposure to this content can be emotionally disturbing and traumatic, especially for those already struggling with mental health issues [20,25].

The majority of participants did not search for specific suicide-related web pages, groups, or profiles. This lack of targeted searching suggests that users may be more open to engaging with a variety of content they encounter in their search results, potentially including information about suicide methods. This underscores the importance of the quality and nature of SRC that appears in search results, as it could have a significant impact on vulnerable individuals. Notably, in our sample, participants who were motivated to engage in SRIU by wanting to search for methods were more likely to search for specific sites. This suggests that these patients are more proactive in their search, potentially indicating higher intent, as previously reported [26].

This study was the first to ask suicidal mental health patients about their interaction with search engines’ “suicide prevention result.” The high prevalence of actively searching for SRC in our sample highlights the importance of web-based prevention efforts, as these searches represent opportunities to reach a significant number of people who may be experiencing a crisis. We found that most participants (85%) have seen some suicide prevention messaging while engaging in SRIU and those who were motivated by help-seeking were more likely to engage with them. The neither harmful nor helpful way the SPR was perceived among participants and higher engagement by those motivated by help-seeking suggest potential shortcomings in its current design. The one-size-fits-all approach of displaying the same SPR regardless of what is being searched and who is searching it seems to be lacking [12,27-29]. To improve the effectiveness of SPR, it would be helpful to target variable messaging based on user demographics and search history, provide different formats such as text, videos, and interactive tools, and create age-appropriate messaging tailored for different age groups.

The importance of timely disclosure of SRIU for creating prevention opportunities has been highlighted in previous research, alongside the lack of routine inquiry about it by clinicians in the United Kingdom [9,30,31]. This study supports these findings from the patients’ perspective, showing that only about 1 in 10 patients in our sample have been asked about SRIU by their clinician. Additionally, it provides a first insight into patients’ self-disclosure of SRIU, revealing that only around a quarter have voluntarily disclosed this information. This is a concerning finding, as clinicians reported relying on patients’ spontaneous disclosure to learn about SRIU [9,31]. Consequently, the low rates of both inquiry and self-disclosure suggest that many opportunities for intervention and exploration of the significance of SRIU are missed. Given these findings, it is crucial for clinicians to take the initiative in asking about SRIU, as this first step can significantly increase the chances of identifying and addressing this behavior, potentially opening up important avenues for intervention and support.

### Strengths and Limitations

One key strength of this study is its large and national sample, which enhances the reliability of our findings; in comparison, the next largest sample for a similar study was 3 times smaller (n=205) [32]. Furthermore, as we delineated the type of SRIU and the motivations behind it, we were able to examine the SRIU characteristics in more detail than in other studies. Additionally, our study is the first to ask about the perception of and interaction with suicide prevention search results in mental health patients who engage in SRIU, a highly vulnerable population. However, several limitations may influence the study’s results. First, our study was conducted in the United Kingdom, and the generalizability of our findings on other nations is limited by national differences in internet infrastructure, suicide prevention resources, and cultural attitudes toward help-seeking. In some regions, limited internet access means that individuals may not rely on web-based sources for suicide-related information, potentially leading to different patterns of information-seeking behavior. Moreover, Google’s OneBox suicide prevention feature and other search engine interventions are not globally uniform, with some countries lacking equivalent prevention tools due to the absence of a national suicide hotline. Therefore, while our findings offer valuable insights into SRIU among mental health patients in the United Kingdom, they may not be fully generalizable to other contexts where internet infrastructure and suicide prevention resources differ. For instance, we did not ask participants about the timing of their SRIU relative to their suicidal ideation or attempts. This prevents us from exploring the direction of the relationship between suicidality and SRIU in the context of time. This study’s cross-sectional design prevents causal conclusions, and participant self-selection introduced biases, with some groups or behaviors potentially being over- or underrepresented. As a result, the findings may not be generalizable to all mental health patients who engage in SRIU. Moreover, as this is a cross-sectional study, we are unable to track potential fluctuations in SRIU over time. Consequently, this research should be seen as a “snapshot” of SRIU among mental health patients. Finally, we inquired about SRIU in general and did not focus on any specific search engine, webpage, or social media. This decision was made due to the constantly changing landscape of the internet, with the aim of improving the generalizability of the findings.

While this study focuses on the characteristics of SRIU itself, a forthcoming study using this dataset will specifically examine the characteristics of individuals who have engaged in SRIU. This will provide a deeper understanding of the demographic, clinical, and suicidality factors associated with engagement in SRIU, complementing the findings presented here.

### Implications for Prevention

Our results highlight the need for consideration of the web-based content available to users, ensuring that those engaging in SRIU are met with helpful and educational resources. The high prevalence of actively searching for SRC via the web suggests that SPR and similar web-based interventions are a first line of defense, and should be improved. For example, a recent study found that internet users with different levels of suicidality respond and engage differently depending on the wording in SPR [29]. This suggests that algorithms displaying SPR should consider tailoring the wording for different groups of web-based users in order to maximize effectiveness, especially given that search engines like Google already possess user data that could enable more targeted prevention strategies [33]. Additionally, advancements in artificial intelligence, now integrated into search engines, are improving the ability to detect nuanced queries, triggering SPR even when searches are not explicitly suicide-related [34]. Once triggered, the prevention potential of SPR seems to lie in its versatility, such as offering both telephone- and text-based services [35]. It may be beneficial to integrate contact information for local crisis services, especially those with different modalities of contact (ie, telephone, text, and web-based), along with educational materials on SRIU, into existing SPRs. These educational materials could provide technical solutions to reduce harmful SRIU, such as resetting their social media accounts if they are being shown harmful SRC, installing browser extensions that block such content, and offering support options instead.

Our finding that more than half of the participants were unaware of how to safely navigate web-based suicide content, highlights the need for continuous education on internet safety. This aligns with Pretorious and colleagues [22] who identified a lack of mental health literacy as a barrier to effective web-based help-seeking. Expanding the reach of resources like the “#chatsafe” guidelines, which promote safer communication about suicidality via the web, by integrating them into existing SPR could be valuable [36,37]. However, these guidelines were designed for young people, and it remains unclear whether they are suitable for adults and mental health patients’ populations or if adaptations are needed.

Apart from integrating these resources via the web, mental health patients can benefit from direct engagement with clinicians who can guide them toward beneficial and help mitigate harmful SRIU. Clinicians should understand this distinction to develop safety plans addressing both suicidal thoughts and behaviors, including SRIU. Additionally, clinicians can signpost patients to verified and high-quality web-based resources on suicidality and mental illness, as well as educational materials on how to navigate the internet in a safer way. As previous research has emphasized, a critical aspect of addressing SRIU in the mental health services setting is the clinician’s direct and nonjudgmental inquiry into whether and why a patient is engaging in such behavior.

### Conclusions

This study underscores the need for both web-based and offline strategies to address SRIU among mental health patients, highlighting key gaps in these areas. Given that searches for SRC via the web represent the most common type of SRIU, there is a need to enhance existing web-based interventions. Currently, these interventions are not perceived as particularly helpful or harmful and are primarily accessed by those already inclined to seek help. Meanwhile, clinicians have untapped potential to reduce harmful and promote beneficial SRIU in their patients, though inquiry and disclosure around SRIU remain rare in clinical settings. Finally, empowering mental health patients with knowledge about safe internet practices should be a shared objective between web-based platforms and mental health service providers.

## Supplementary material

10.2196/70458 Multimedia Appendix 1 Questionnaire.

## Abbreviations

SPR: suicide prevention result
SRC: suicide-related content
SRIU: suicide-related internet use
VAS: visual analog scale
