# Supplementary material for: Exploring Suicide-Related Internet Use Among Suicidal Mental Health Patients in the United Kingdom: Cross-Sectional Questionnaire Study
Source: JMIR Ment Health. 2025 Jul 8;12:e70458. doi: 10.2196/70458 (PMC12262929; doi:10.2196/70458)
Supplement: Multimedia Appendix 1 [file mental-v12-e70458-s001.docx]

**Online survey of people in contact with mental health services with suicidal thoughts and/or behaviours who use the Internet**
 
  **Please click** [this link](http://documents.manchester.ac.uk/display.aspx?DocID=66986) **to read and download the participant information sheet.**

 **Am I suitable to take part?**
You can take part if you are:
 
a) aged **18 or above**,

 b) have been **in contact with secondary mental health services within the past 12 months**,

 c) have experienced **suicidal thoughts/behaviours/attempts in the past 12 months**,

 d) **use the Internet,**

 e) **live in the UK,** and
 
f) have a **good command of English language**.

- Please select this option to confirm you have read the PiS

Help in a crisis
 *We do not provide treatment or care service or advice for those in crisis. If you are in crisis or feeling suicidal, we urge you to seek help:*

 • From your mental health provider
 • From your general practitioner
 • From your local hospital emergency department
 • Through a telephone helpline service
 • By discussing your problem with a friend or colleague

 Online help
 *Some useful websites that may be of help are listed below. Click on the link to go directly to the website:*

 • [NHS help for suicidal thoughts](https://www.nhs.uk/mental-health/feelings-symptoms-behaviours/behaviours/help-for-suicidal-thoughts/)
 • [Samaritans](https://www.samaritans.org/how-we-can-help/contact-samaritan/)
 • [Papyrus](https://www.papyrus-uk.org/)
 • [Turning point (crisis point helpline)](https://www.turning-point.co.uk/services/mental-health/crisis-support.html)
 • [Childline](https://www.childline.org.uk/get-support/)
 • [Shout](https://giveusashout.org/get-help/)
 • [Lesbian and Gay Foundation](https://lgbt.foundation/howwecanhelp)
 • [NHS online 111](https://www.nhs.uk/nhs-services/urgent-and-emergency-care-services/when-to-use-111/)

 Phone helplines
 *Useful telephone numbers:*

 • find your local NHS 24/7 mental health crisis helpline:
   https://www.nhs.uk/ ‘Find a local NHS urgent mental health helpline’
 • Samaritans: **116 123**
 • Papyrus (prevention of young suicide (under 35)): **0800 068 4141**
 • Crisis Point: **0161 839 5030**
 • Childline: **0800 1111**
 • Lesbian and Gay helpline: **0161 235 8000 (6.00 - 10.00 pm)**
 • NHS: **111**

- Please select this option to consent to participate in this study.

Inclusion criteria questions (2)

1. Have you been in contact with secondary mental health services* in the past 12 months?

**Examples of secondary mental health services are hospitals, community mental health teams (CMHTs), crisis resolution and home treatment teams (CRHTs), assertive outreach teams and early intervention teams.*

- No
- Yes

2. Have you experienced suicidal thoughts and/or engaged in suicidal behaviour in the past 12 months?

- No
- Yes

If either is ‘No’ the following is displayed + End Of Survey:

You do not meet the criteria for this study, but thank you for your interest.

Section 1: Sociodemographic questions (8)

1. Your age

- <18
- 18-24
- 25-34
- 35-44
- 45-54
- 55-64
- 65-74
- 75<

If <18 is selected the following is displayed + End Of Survey:

As you are aged under 18, you do not meet the criteria for this study. Thank you for your interest. If you struggle with suicidal thoughts and/or behaviours please contact: Childline – helpline for children and young people aged under 19: 0800 1111
(the number will not show up on your phone bill)
Papyrus- prevention of young suicide (under 35): 0800 068 4141

If over 18 selected participant proceeds:

*Note: please be mindful not to fill any ‘open text field’ categories with personal identifiable information, such as your email address or last name.*

2. Your gender:

- Male
- Female
- Prefer not to say

3. Does your gender identity match the sex assigned to you at birth?

- Yes
- No, I am transgender
- No, I am non-binary
- Prefer not to say
- Let me write... (open text)

4. How would you describe your ethnicity?

- Arab/Middle Eastern
- Asian/Asian British
- Black/African/Caribbean/Black British
- Mixed/multiple ethnic group
- White/White British/White Irish
- Prefer not to say
- Let me write... (open text)

5. Your sexual orientation

- Heterosexual
- Lesbian/Gay man/Gay
- Bisexual
- Queer
- Questioning
- Prefer not to say
- Let me write... (open text)

6. Your education level

- No qualifications
- GCSEs/O Levels
- A or AS Levels / Highers or Advanced Highers
- Vocational or work-based qualification (e.g. NVQs, HNC / D)
- Undergraduate degree
- Postgraduate qualification (e.g., Certificate, Diploma, Masters, PhD)
- Prefer not to say
- Let me write… (open text)

7. Your employment status

- In paid employment (including part-time, self-employed)
- Unemployed
- Homemaker
- Full-time student
- Retired
- Government training scheme/Apprenticeship/Training scheme
- On long-term sick leave
- Prefer not to say
- Let me write... (open text)

8. Relationship status

- Single
- Married / co-habiting / civil partnership
- In a relationship (not co-habiting)
- Separated / divorced
- Widowed
- Prefer not to say
- Let me write... (open text)

2. Clinical history (5)

*Note: please be mindful not to fill any ‘open text field’ categories with personal identifiable information, such as your email address or last name.*

1. Have you been an **in-patient** within secondary mental health services in the past 12 months?

- No
- Yes
- Prefer not to say

2. Did you receive any of the following diagnoses (within the last 12 months or before)?

|  | In the last 12 months (1) | At any point before (2) |
| --- | --- | --- |
| None |  |  |
| Schizophrenia or other primary psychotic disorders |  |  |
| Drug-induced psychotic disorder |  |  |
| Bipolar affective disorder |  |  |
| Depressive disorder |  |  |
| Anxiety disorder/Phobia/Panic disorder/Obsessive-compulsive disorder (OCD) |  |  |
| Post-traumatic stress disorder (PTSD) |  |  |
| Eating disorder |  |  |
| Alcohol dependence / misuse |  |  |
| Drug dependence / misuse |  |  |
| Personality disorder |  |  |
| Adjustment disorder |  |  |
| Learning disability |  |  |
| Autistic spectrum disorder |  |  |
| ADHD |  |  |
| Prefer not to say |  |  |
| Let me write.. (open text) |  | |

3. Have you been prescribed any medication for your mental health condition (e.g. antidepressants/antipsychotics) in the past 12 months?

- No
- Yes
- Prefer not say

4. Please select the option 'Always'

- Never
- Sometimes
- About half the time
- Most of the time
- Always

5. In the past 12 months, have you been satisfied with the care received from your secondary mental health provider? Select what is most relevant to you.

|  | Very dissatisfied | Neither satisfied nor dissatisfied | Very satisfied |
| --- | --- | --- | --- |

|  | 0 | 1 | 2 | 3 | 4 | 5 | 6 | 7 | 8 | 9 | 10 |
| --- | --- | --- | --- | --- | --- | --- | --- | --- | --- | --- | --- |

|  | 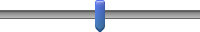 |
| --- | --- |

Section 3: Suicidal thoughts and behaviours (11)

*Note: please be mindful not to fill any ‘open text field’ categories with personal identifiable information, such as your email address or last name.

We understand that some of the following questions might be distressing. You can skip any questions you don’t want to answer, or stop completing the survey at any time.*

1. In the last 12 months, have you had thoughts of suicide?

- No
- Yes
- Prefer not to say

If anything but ‘Yes’ is selected, participant is forwarded to question 5.

2. If yes, how often have you had thoughts of suicide?

- Every day or almost every day
- 2-5 times a week
- Once a week
- Once a month
- Every few months
- Just once

3. How would you describe the average intensity of these thoughts? Select a response that is most relevant to you.

|  | Not at all intense | Quite intense | Very intense |
| --- | --- | --- | --- |

|  | 0 | 1 | 2 | 3 | 4 | 5 | 6 | 7 | 8 | 9 | 10 |
| --- | --- | --- | --- | --- | --- | --- | --- | --- | --- | --- | --- |

|  | 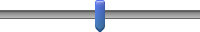 |
| --- | --- |

4. Have you told anyone about your thoughts of suicide?

- No
- Yes, friends and/or family
- Yes, a mental health professional
- Yes, someone else… (open text)

5. In the last 12 months, have you attempted suicide/tried to take your own life?

- No
- Yes
- Prefer not to say

If anything but ‘Yes’ is selected, participant is forwarded to question 10.

6. If yes, how many times?

- Once
- Twice
- Three or more times

7. How much planning was involved in your suicide attempt?
*Select a response that is most relevant to you.*

|  | Not planning at all | Some planning | A lot of planning |
| --- | --- | --- | --- |

|  | 0 | 1 | 2 | 3 | 4 | 5 | 6 | 7 | 8 | 9 | 10 |
| --- | --- | --- | --- | --- | --- | --- | --- | --- | --- | --- | --- |

|  | 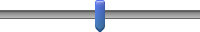 |
| --- | --- |

8. Did you tell anyone you were planning to attempt suicide?

- No
- Yes, friends and/or family
- Yes, a mental health professional
- Yes, someone else (open text)
- Prefer not to say

9. Did you receive any medical assistance following your suicide attempt?

- No
- Yes
- Prefer not to say

10. Did any of the following life events contribute to your suicidal thoughts and/or behaviours in the past 12 months? Please select all that apply

- Relationship difficulties (breakup, separation, divorce)
- Serious financial difficulties
- Gambling
- Alcohol misuse
- Drug misuse
- Criminal charges or conviction
- Violence as a victim
- Violence as a perpetrator
- Bereavement
- Physical illness
- COVID-19 related issues (e.g. long COVID)
- Menopause
- Fertility problems or concerns
- Insomnia
- Prefer not to say
- Let me write… (open text)

11. How much support from friends/family/community have you received for your mental health in the past 12 months?
*Select a response that is most relevant to you.*

|  | Not a lot of support | Some support | Lots of support |
| --- | --- | --- | --- |

|  | 0 | 1 | 2 | 3 | 4 | 5 | 6 | 7 | 8 | 9 | 10 |
| --- | --- | --- | --- | --- | --- | --- | --- | --- | --- | --- | --- |

|  | 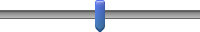 |
| --- | --- |

4. Suicide-related internet use (15)

*Note: please be mindful not to fill any ‘open text field’ categories with personal identifiable information, such as your email address or last name.

We understand that some of the following questions might be distressing. You can skip any questions you don’t want to answer, or stop completing the survey at any time.*

**Suicide-related internet use is any use related to your thoughts, feelings and behaviours connected to suicide. This kind of use can be, for example, expressing suicidal feelings or thoughts on social media or using the internet to get help and support when feeling suicidal.**

1. In the past 12 months, have you: (please select all that apply)

- Searched for suicide related content/information (e.g. browsing the Internet or social media) because of your own suicidal thoughts and feelings
- Created suicide related content/information (e.g. blogging or posting pictures)
- Engaged with (e.g. commented or reposted) suicide-related content
- Accidentally came across suicide-related content while browsing internet/social media
- Used the Internet to interact/connect with others because of your own suicidal thoughts and feelings
- None of the above

If ‘None of the above’ is selected, the following message is displayed + End Of Survey:

Thank you so much for your interest and participation! This is the end of the survey

2. What was your suicide-related Internet use most commonly motivated by? *(Please select all that apply)*

- Seeking information on support/help for my own mental health (e.g. Samaritans, NHS web pages)
- Seeking information on methods of suicide/harming oneself
- Seeking support from friends/peers
- Interacting with celebrity bloggers/influencers
- Engaging in discussions about suicide/harming oneself (e.g. forums, Twitter)
- Sharing personal experiences of suicidality (e.g. on social media)
- Researching medication (e.g. side effects) that you have been taking for your mental health
- Curiosity about suicide
- Raising awareness and/or campaigning for suicide prevention
- Let me write... (open text)

3. If you searched for suicide-related content using web browsers like Google, which search terms have you used? *(if you can remember)*

(open text)

4. Were you looking for a specific suicide-related website/profile/account/group?

- No
- Yes

5. In the past 12 months, how often were you using the Internet for suicide-related purposes?

- Every day or almost every day
- 2-5 times a week
- Once a week
- Once a month
- Every few months
- Just once

6. How helpful or harmful was your suicide-related Internet use? 
*Select a response that is most relevant to you.*

|  | Very helpful (e.g. relieved my distress significantly) | Neither helpful nor harmful | Very harmful (e.g. increased my distress significantly) |
| --- | --- | --- | --- |

|  | 0 | 1 | 2 | 3 | 4 | 5 | 6 | 7 | 8 | 9 | 10 |
| --- | --- | --- | --- | --- | --- | --- | --- | --- | --- | --- | --- |

|  | 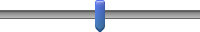 |
| --- | --- |

7. Have you seen any suicide prevention messaging during your suicide-related Internet use (e.g. Samaritans helpline notices)?

- No
- Yes
- Not sure/cannot recall

If ‘Yes’ is selected, questions 8 and 9 are displayed; if anything but ‘Yes’ is selected, participant is forwarded to question 10.

8. If you do recall seeing any, have you ever interacted with the interventions?
*(e.g. followed the links, called the helpline numbers provided)*

- No
- Yes
- Prefer not to say

9. If you interacted with the intervention(s), did you find them helpful?
*Select a response that is most relevant to you.*

|  | Very helpful (e.g. relieved my distress significantly) | Neither helpful nor harmful | Very harmful (e.g. increased my distress significantly) |
| --- | --- | --- | --- |

|  | 0 | 1 | 2 | 3 | 4 | 5 | 6 | 7 | 8 | 9 | 10 |
| --- | --- | --- | --- | --- | --- | --- | --- | --- | --- | --- | --- |

|  | 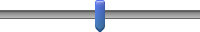 |
| --- | --- |

10. Do you know where to find more information on how to use the Internet safely when searching for and accessing suicidal content?

- No
- Yes
- Not sure

11. Have you told your mental health professional (e.g. nurse, psychiatrist, psychologist) about your suicide-related Internet use?

- No
- Yes
- Prefer not to say

12. Have you been asked by your mental health professional (e.g. nurse, psychiatrist, psychologist) about any suicide-related Internet use?

- No
- Yes
- Not sure
- Prefer not to say

If ‘Yes’ is selected question 13 is displayed, if anything but ‘Yes’ is selected, question 14 is displayed.

13. If yes, what advice did your mental health professional give you?

(open text)

14. If you did not talk to your mental health professional about your suicide-related Internet use, why not?

(open text)

15. Is there any other information you want to provide regarding suicide-related Internet use? Any insights and lived-experience you may be willing to share is extremely valuable.
We appreciate your time and how difficult parts of the questionnaire may have been for you.

(open text)

5. End Of Survey

The following is displayed at every exit from the survey

**Help in a crisis**

*If you are in crisis or feeling suicidal, we urge you to seek help:*
• From your general practitioner
• From your local hospital emergency department
• Through a telephone helpline service
• By discussing your problem with a friend or colleague

Online help
*Some useful websites that may be of help are listed below. Click on the link to go directly to the website:*
• [NHS help for suicidal thoughts](https://www.nhs.uk/mental-health/feelings-symptoms-behaviours/behaviours/help-for-suicidal-thoughts/)
• [Samaritans](https://www.samaritans.org/how-we-can-help/contact-samaritan/)
• [Papyrus](https://www.papyrus-uk.org/)
• [Turning point (crisis point helpline)](https://www.turning-point.co.uk/services/mental-health/crisis-support.html)
• [Childline](https://www.childline.org.uk/get-support/)
• [Shout](https://giveusashout.org/get-help/)
• [Lesbian and Gay Foundation](https://lgbt.foundation/howwecanhelp)
• [NHS online 111](https://www.nhs.uk/nhs-services/urgent-and-emergency-care-services/when-to-use-111/)

Phone helplines
Useful telephone numbers:
• find your local NHS 24/7 mental health crisis helpline:
  https://www.nhs.uk/ ‘Find a local NHS urgent mental health helpline’
• Samaritans: **116 123**
• Papyrus (prevention of young suicide (under 35)): **0800 068 4141**
• Crisis Point: **0161 839 5030**
• Childline:**0800 1111**
• Lesbian and Gay helpline: **0161 235 8000** (6.00 - 10.00 pm)
• NHS: **111**
